# Exploration of Internal and External Factors of Swimmers’ Performance Based on Biofluid Mechanics and Computer Simulation

**DOI:** 10.3390/ijerph18126471

**Published:** 2021-06-15

**Authors:** Yifan Liu, Gang Lu, Junke Chen, Qigang Zhu

**Affiliations:** 1 Department of Electrical Engineering & Information Technology, Shandong University of Science and Technology, Jinan 250031, China; 201803204418@sdust.edu.cn (Y.L.); 201903204415@sdust.edu.cn (G.L.); 2 Department of Finance and Economics, Shandong University of Science and Technology, Jinan 250031, China; 201903104102@sdust.edu.cn

**Keywords:** swimming, biomechanics, biofluid mechanics, swimming speed representation model, drag effective dynamic model, computer simulation

## Abstract

The purpose of this study is to explore the influence of different swimming strokes on the performance of swimmers and the resistance of each part from the perspective of hydrodynamics. In this paper, the influence of internal and external factors on the swimming speed is analyzed comprehensively and meticulously from the macro and micro perspectives. In the macroscopic part, the swimming speed representation model is established, and the validity of the model is further verified by the analysis of experimental data and hydrodynamic equations. In the microscopic part, we carefully analyzed details such as the opening angle of the palm, the timing of the arm and leg and the angular velocity of each link of the human body. Combined with computer simulation, stereo modeling and numerical analysis are carried out, and the best scheme FOR how to cooperate with each part of the body in swimming is given.

## 1. Introduction

**Figure 1:**
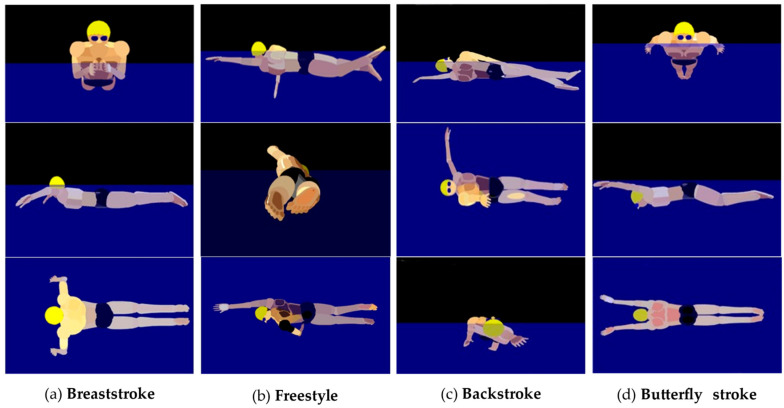
Front view, side view and top view of the four strokes. (**a**) Breaststroke action view. (**b**) Freestyle stroke attempts. (**c**) Backstroke action view. (**d**) Butterfly action view.

Swimming, as a sport loved by the majority of people, can strengthen the body [1] and is highly regarded by athletes [2]. Countries around the world have also trained a large number of outstanding athletes, and they have also achieved excellent results [3] in international competitions [4]. Swimming is divided into four categories according to different swimming styles [5]: breaststroke [6], butterfly [7], backstroke [8] and freestyle [9]. Different swimming styles have different techniques and methods, as shown in Figure 1. Each stroke also has its own type of training.

The swimmer’s speed depends on the relationship between the swimmer’s resistance [10] and fluid dynamics. Both of these forces block the swimmer’s momentum [11]. Therefore, while training should enhance the propulsion force, it must also reduce the resistance in order to move forward better. Given the increasing competitiveness and skill [12] of swimmers, it is important to understand more about the changes in swimmers’ speeds caused by hydrodynamics not only in terms of performance, but also in terms of technology [13] so that professional swimmers and swimmers can train better.

Nowadays, with the increasing competitiveness and participation of swimmers, it is necessary to further explore the variation of swimmers’ speeds caused by various factors, including not only the influence of external factors, but also the changes in the details of the swimmer’s own movements. In the field of studying swimmers’ speed improvement, previous scholars have made extensive preparations [14]. Nicol et al. [15] explored the effect of turn technique on swimmers from the perspective of bio-hydrodynamics. In the field of bio-hydrodynamics, some scholars set out to analyze the details of body movements and explore the influence of the movements of the trunk and legs on the swimming speed [16]. Ruiz-Navarro et al. explored the relationship between underwater fluctuations and kinematics [17], which enabled us to understand the impact of the swimming environment. Other work also mainly focuses on the analysis of swimmers themselves [18] or the swimming environment [19]. Other works analyze a part, such as the arms [20] or skin [21]. Although everyone has contributed a lot of work in this field, no one has conducted a coherent study from the external macro perspective to the micro perspective of the action details. Our research aims to fill this gap.

In order to fill this gap, this paper analyzes the influence of the external environment on the swimmer and the influence of the details of the swimmer’s own movements on swimming progress from the external macro and internal micro perspectives. Finally, in the conclusion, according to the results of this study, we give the adjustment scheme for the athletes’ training.

The main contributions are as follows:

1. From the perspective of fluid mechanics, a swimming speed representation (S2R) model based on biological fluid dynamics is established. This model can macroscopically reflect various factors that affect a swimmer’s speed.
2. We improve the TOPSIS [22] model and combine it with the S2R model to find out the best swimming style for speed and thrust as a further direction of exploration.
3. The Technology for Order Reference by Security to an Ideal Solutions (TOPSIS) method was first proposed by C.L. Hwang and K. Yoon in 1981, and the TOPSIS method sorts the proximity of finite evaluation objects to idealized targets, evaluating the relative advantages and disadvantages in existing objects. The TOPSIS method is a sorting method that approximates to an ideal solution which requires only the monotonically increasing (or decreasing) properties of each utility function. The TOPSIS method is a common effective method in multi-objective decision analysis, also known as the solution distance method.
4. Based on the macroscopic analysis, the changes of swimming resistance caused by the detailed movements of the body, such as the hand, arm, leg and trunk, are analyzed by computer simulation and modeling. Finally, in the conclusion, it provides detailed guidance and suggestions for swimming enthusiasts, especially athletes’ training.

## 2. Related Works

It is generally believed that swimming speed is mainly determined by the two most direct factors of stroke (R) and frequency (L); specifically, speed S = R × L [23]. Frequency refers to the number of periodic swimming actions per unit of time. Stroke refers to the displacement distance generated by each swimming cycle. The influencing factors of the stroke R mainly depend on the driving force and resistance in the water. We use the work formula to combine the stroke with the driving force and resistance and use the classic N-S equation [24] of fluid mechanics to express the resistance and driving force. In the process of representation, we found that the cross-sectional area perpendicular to the direction of water flow is an important parameter. We use this as a breakthrough to study the connection between it and the swimming style. We also combine the fluid dynamics model with the energy conversion model to find the expression of the water flow velocity in the N-S equation.

### 2.1. Hydrodynamic Model

Mechanics is the science of studying the mechanical motion of objects. It is the study of macroscopic motion that can be seen by the eyes, and it does not involve the motion of microscopic molecules and atoms. Fluid mechanics is a branch of mechanics. It takes fluid as the object of study, including liquid [25] and gas. It mainly studies the interaction and reaction between fluids and fluids or fluids and solids. There are three research methods in fluid mechanics: theory, calculation and experimentation. According to different research methods, fluid mechanics can be divided into theoretical fluid mechanics [26], experimental fluid mechanics [27] and computational fluid mechanics. The study of theoretical fluid mechanics generally establishes theoretical models through scientific approximation and then uses mathematical methods to obtain theoretical results, thereby clearly and universally revealing the internal laws of material movement. The research of experimental fluid mechanics mainly involves conducting model experiments or physical experiments in experimental equipment such as wind tunnels and water tanks. Its characteristic is that it can be observed under the same conditions since the question being studied or the answer is the same, so the conclusions drawn from the experiment are reliable. Computational fluid dynamics is a combination of modern fluid mechanics, numerical mathematics and computer science, and it is a fringe science. This model will be applied in our work.

### 2.2. CFD

CFD [28] is the abbreviation of computational fluid mechanics, which is a new interdisciplinary subject integrating fluid mechanics and computer science. The approximate solution of the governing equation of fluid is obtained by using the fast computing power of computers. CFD emerged in the 1960s. With the rapid development of computers after the 1990s, CFD has developed rapidly and gradually become an important methods of product development together with experimental fluid mechanics. It takes the computer as a tool and uses various discrete numerical methods to carry out numerical experiments, computer simulations and analysis on various problems of modern fluid mechanics so as to solve various practical problems and reveal new physical phenomena. The application of this tool will be reflected in our work.

### 2.3. Energy Conversion Model

Energy conversion refers to the conversion of energy forms, such as fuel, which can be converted into heat energy through combustion, and heat energy can be converted into mechanical energy through a heat engine. Energy is changing from one form to another and from one object to another, and the amount of energy is constant in the process of energy conversion and energy transfer. This model will be applied in our work.

### 2.4. TOPSIS

The TOPSIS method, also known as the ideal solution method, is an effective multi-index evaluation method. In this method, the positive ideal solution and negative ideal solution of the evaluation problem are constructed, namely the maximum and minimum value of each index, and the relative closeness degree of each solution to the ideal solution is calculated—that is, the degree close to the positive ideal solution and far away from the negative ideal solution—to sort the schemes so as to select the optimal scheme. This model will be used to analyze the example.

## 3. Methods and Materials

When analyzing the swimmer’s forward movement in the water, propulsion and resistance are important indicators used to evaluate the athlete’s travel speed. For these two indicators, fluid mechanics is undoubtedly an effective means. Therefore, our research will be carried out on the basis of fluid mechanics.

In the process of using the classical N-S equations of fluid mechanics to solve the force and resistance, we need to determine the cross-sectional area A perpendicular to the direction of the flow. We can model A by combining the movements of different strokes. Based on this method, the speed and thrust of the four swimming strokes are sorted by the real experimental data, and an improvement of the swimmer’s movement is proposed.

### 3.1. Macro Perspective Analysis

#### 3.1.1. Construction of Swimming Speed Representation System

First, let us introduce hydrodynamic force. Since the water pressure on various parts of the human body in the water is different, the hydrodynamic force acting on some parts of the human body is opposite that of the swimming direction; that is to say, resistance is generated. The hydrodynamic force acting on other parts is the same as the swimming direction; that is, propulsion is generated. In this sense, the hydrodynamic force acting on the entire human body is the resultant force of the dynamic resistance $F_{d}$ and propulsion $F_{l}$ [19]—that is, their algebraic sum—which can be written as the following formula:

(1) $$F=F_{d}+F_{l}$$

According to the situation analysis, the average speed of completing a swimming action V can be obtained:

(2) V=L×B

where *L* represents the stroke of one movement cycle and *B* represents the number of swimming periodic movements per unit of time:

(3) $$V=\frac{L}{t}$$

where *t* represents the time of an action cycle.

The formula for the work done by the thrust and resistance to the swimmer is

(4) Z=F·L

Therefore, *L* can be expressed by Equation (4) as the following formula:

(5) $$L=\frac{Z}{F}$$

We assume that the athlete swims with full force before and after the additional small disturbance is applied, and that the swimmer’s body shape is basically unchanged. Therefore, the drag coefficient is constant, and the resistance should be the same for the two advances. The derivation of the forward and backward thrust resistance is as follows:

(6) $$F_{d}=\frac{1}{2}C_{d}\cdot \rho \cdot A\cdot V^{2}$$

(7) $$F_{l}=\frac{1}{2}C_{l}\cdot \rho \cdot A\cdot V^{2}$$

where $C_{d}$ and $C_{l}$ are the resistance coefficients under the corresponding conditions. Swimming involves a complex physiological process [29]. The human body relies on aerobic metabolism [30] and anaerobic metabolism to provide the energy needed for exercise. The energy storage of the human body can be expressed by Equation (8):

(8) E=K−Z−N−r

where *K* is the energy generated by metabolism, Z=Buv is the horizontal propulsion energy, which provides energy for swimming movement, *N* is the non-propulsion energy, which enables a certain mass of water to have kinetic energy (i.e., speed) and *R* is the energy consumed in the form of heat energy [31].

In Equations (6) and (7), it is assumed that the density of a fixed pool does not change, and the swimming speed V can be expressed in Equation (9) simultaneously:

(9) $$V=\frac{\sqrt{2\left(Z+N\right)}}{m}$$

Together with all the above formulas, the expression model of the swimming speed is established, where P represents the density of water. The m stands for the athlete’s quality:

(10) $$V=\frac{Zm}{t\rho A\left(Z+N\right)\left(C_{d}+C_{l}\right)}$$

Equation (10) is the swimming speed representation model (S2R), which covers the various influencing factors that will affect the swimmer’s speed from a macro perspective.

#### 3.1.2. Improved Distance Method of Superior and Inferior Solutions

Since the cross-sectional area perpendicular to the direction of the water flow changes with the change of the action, we used the model in Equation (10) in combination with the influencing factors to determine the advantages and disadvantages of various swimming styles. We measured the actual experimental data of 15 swimmers as shown in Table 1. Table 2 shows some representative parameters collected from athletes of different swimming styles during the swimming process. (The specific measurement method is explained in the experimental part.) In order to make the sample data more representative, we chose the sample method as follows: S1–S15 are 15 athletes, the average height was 1.85 ± 0.1 m and the average weight was 72 ± 13 kg. In order to unify the variables and simplify the complexity of the model, we chose to collect the parameters of the right leg to represent the limbs. The process was as follows.

The second, third and fourth columns show the joint angle or link angle corresponding to the maximum moment of the limb, respectively. It should be noted that the link angle refers to the angle between the longitudinal axis of the link (from the far side to the near side) and the vertical axis.

In order to better homogenize the attributes and positive indicators, we declared the following indicators:

1. Miniaturization index:

(11) $$x^{\prime }=\frac{1}{x},\;\;\;\;\;\left(x>0\right)$$

Alternatively, this can be expressed as

(12) $$x^{\prime }=M-x$$

2. Intermediate index:

(13) $$x^{\prime }=\{\begin{array}{c} 2\frac{x-m}{M-m},\;\;\;\;\;\;\;m\leq x\leq \frac{1}{2}\left(M+m\right)\\ 2\frac{M-x}{M-m},\;\;\;\;\;\;\;\frac{1}{2}\left(M+m\right)\leq x\leq M \end{array}$$

3. Interval indicators (the value of the expected indicator is best when falling into a certain interval):

(14) $$x^{\prime }=\{\begin{array}{c} 1-\frac{a-x}{a-a^{*}},\;\;\;\;\;\;\;x<a\\ 1,\;\;\;\;\;\;\;a\leq x\leq b\\ 1-\frac{x-b}{b^{*}-b},\;\;\;\;\;\;\;x>b \end{array}$$

4. Construction of the normalized initial matrix:

(15) $$X=\left[\begin{array}{ccc} x_{11} & \cdots & x_{1m}\\ \vdots & \ddots & \vdots \\ x_{n1} & \cdots & x_{nm} \end{array}\right]$$

5. Constructing a weighted canonical matrix and vector normalizing the attributes (i.e., each column element is divided by the norm of the current column vector, measured using the cosine distance):

(16) $$z_{ij}=\frac{x_{ij}}{\sqrt{\sum_{i=1}^{n}x_{ij}^{2}}}$$

6. Getting the normalized matrix after normalization:

(17) $$Z=\left[\begin{array}{ccc} z_{11} & \cdots & z_{1m}\\ \vdots & \ddots & \vdots \\ z_{n1} & \cdots & z_{nm} \end{array}\right]$$

7. Getting the normalized matrix after normalization.

The optimal scheme Z^+^ consists of the maximum value of the elements in each column of Z:

(18) $$Z^{+}=\left(max\left\{z_{11},z_{21},\cdots ,z_{n1}\right\},max\left\{z_{12},z_{22},\cdots ,z_{n2}\right\},\cdots ,max\left\{z_{1m},z_{2m},\cdots ,z_{nm}\right\}\right)=\left(Z_{1}^{+},Z_{2}^{+},\cdots ,Z_{m}^{+}\right)$$

8. The worst solution Z^−^ consists of the minimum value of each column of elements in z:

(19) $$Z^{-}=\left(min\left\{z_{11},z_{21},\cdots ,z_{n1}\right\},min\left\{z_{12},z_{22},\cdots ,z_{n2}\right\},\cdots ,min\left\{z_{1m},z_{2m},\cdots ,z_{nm}\right\}\right)=\left(Z_{1}^{-},Z_{2}^{-},\cdots ,Z_{m}^{-}\right)$$

9. The approximate degree of each evaluation object to the optimal and the worst scheme was calculated:

(20) $$D_{i}^{+}=\sqrt{\overset{m}{\underset{j=1}{\sum }}\omega_{j}{\left(Z_{j}^{+}-z_{ij}\right)}^{2}}D_{i}^{-}=\sqrt{\overset{m}{\underset{j=1}{\sum }}\omega_{j}{\left(Z_{j}^{-}-z_{ij}\right)}^{2}}$$

10. The degree of closeness between each evaluation object and the optimal scheme was calculated:

(21) $$C_{i}=\frac{D_{i}^{-}}{D_{i}^{+}+D_{i}^{-}}$$

According to the data in Table 2, through the analysis of Equations (18) and (21), the speed scores of the 15 athletes were obtained. Their scores are ranked as shown in Table 3.

The data in the table shows that the overall performance score of the freestylers was better than the other swimming styles. Therefore, the details of freestyle athletes’ movements, such as the timing of arm and leg coordination, the angular velocity of the trunk and the angular velocity of the upper and lower legs, were the most instructive.

We combined these results with the classical N-S equation of fluid mechanics, Equations (6) and (7). Based on the experimental data in Table 1, the improved solution distance method was used to calculate the $F_{d}$ scores of all athletes. Then, the players were sorted according to their scores, and the results are shown in Table 3.

The data in Table 4 shows that the thrust generated by the athletes using the breaststroke was better than that of other swimming styles. Therefore, in terms of power, swimmers can learn from the power details of the breaststroke in training.

At this point, we had a macro understanding of how to improve the performance of swimmers. In terms of deeper details, how should athletes train? We will discuss in detail the microscopic direction of swimmers.

### 3.2. Micro Perspective Analysis

In order to explore the swimming posture of athletes more comprehensively, we analyzed the factors that affect the swimming speed from the micro point of view of the human body. This part mainly elaborates on the influence of the opening angle of the fingers, the timing of coordination between the arms and legs and the angular velocity of the link (trunk, thigh and leg) on the swimming stroke efficiency.

#### 3.2.1. Establishment of the Geometric Model of the Palm Angle

The influence of the change of the opening angle of the finger on the projected cross-sectional area of the swimming palm perpendicular to the direction of the incoming flow was numerically simulated by means of computational fluid dynamics (CFD) software fluent [32] (software distribution company: Ansys, with the global headquarters located in Canonsburg, PA, USA). The flow of water around the hand and finger at different velocities, different opening angles of the fingers and different angles of attack of the hand were numerically simulated so as to calculate the resistance. Then, the corresponding resistance coefficient could be obtained, and the trend of change could be discussed.

**Figure 2:**
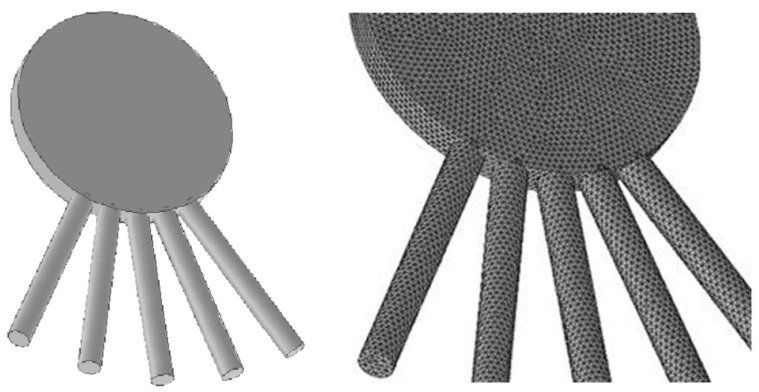
The geometric model of the palm and its surface network.

According to the similitude principle of fluid mechanics, the resistance of a hand in still water is equivalent to that of an incoming flow with the same velocity without moving the hand. We studied the influence of the separation angle of the five fingers on the propulsive resistance by numerical simulation. The geometric simulation diagram of the palm is shown in Figure 2.

The resistance value of the palm’s surface can be obtained by a trigonometric function, and then the projection area A of the palm in the direction of the water flow can be obtained. Since the tension angle between the five fingers is limited, four cases of 15°, 20°, 25° and 30° were taken into consideration. Combined with Equations (18) and (19), we established the effective dynamic model of the stroke:

(22) $$A_{h}=S_{h}\sin\theta$$

(23) $$U_{hand}=\frac{PA_{h}V^{2}}{2F_{d}}$$

where $F_{d}$ is the resistance, V is the incoming flow velocity and $p=998.2\;Kg/m^{3}$ is the density of water. Using the difference equation, the area of the palm was approximately $0.015m^{2}$ when the angle was 90°. The calculated results are shown in Table 5.

It can be seen from the numerical results that the greater the speed is, the greater the resistance is when the angle of the same finger is opened. However, at the same speed, the resistance value did not increase when the angle of the finger was increased; rather, it decreased. Through the change of the drag coefficient, it can be found that no matter how big the corresponding opening angle was, the corresponding drag coefficient showed a downward trend with the increase in velocity, and the change was sharp at a low velocity but gentle at a high velocity.

**Figure 3:**
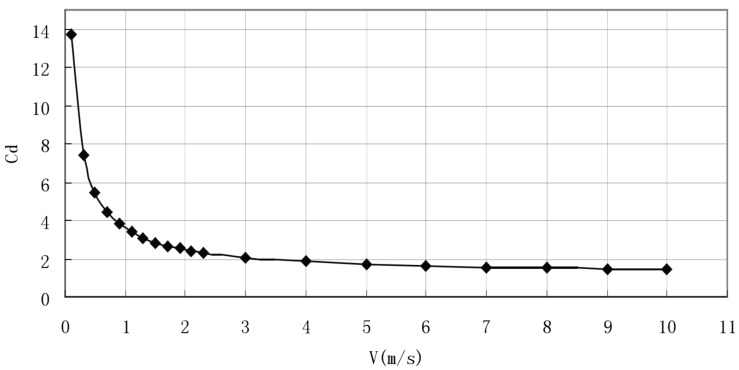
Curve of resistance coefficient changing with velocity. The abscissa is the swimmer’s speed (v), and the ordinate is its resistance coefficient ($C_{d}$).

In order to show more clearly whether the change trend of the resistance coefficient was still decreasing in a larger speed range and whether the change became more and more gentle, we visualized the data to obtain the resistance coefficient [33] with the speed (0.1 ~ 10m/s). The changes are shown in Figure 3.

It can be seen from Figure 3 that when the opening angle was 90° and the finger opening angle was 15°, the drag coefficient still showed a monotonous decreasing trend with the large-scale change of the incoming flow speed, and it decreased more gently as the speed increased. The previous observations are consistent.

**Figure 4:**
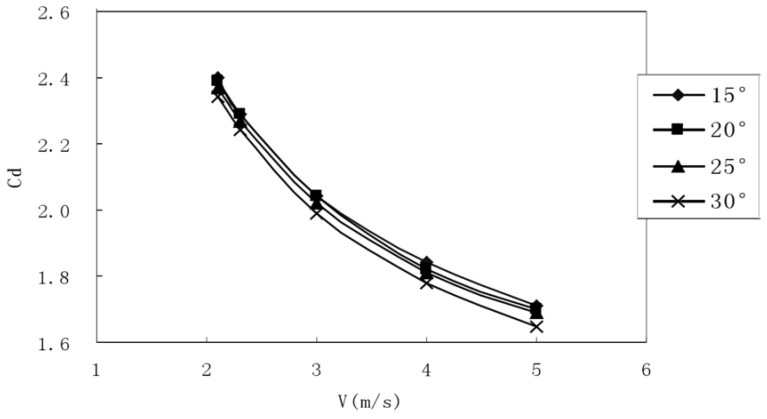
Resistance coefficients corresponding to different finger opening angles (opening angle: 90°). The abscissa is the swimmer’s speed (v), and the ordinate is its resistance coefficient ($C_{d}$). The angle marked in the legend is the palm’s angle of attack.

**Figure 5:**
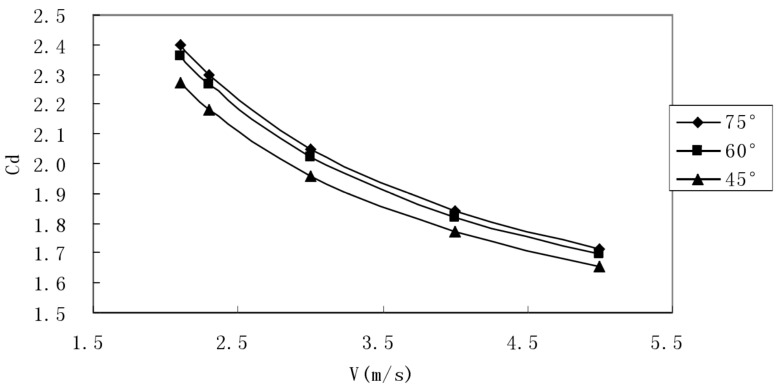
Drag coefficients corresponding to different tension angles (tension angle: 15°). The abscissa is the swimmer’s speed (v), and the ordinate is its resistance coefficient ($C_{d}$). The angle marked in the legend is the palm’s angle of attack.

As shown in Figure 4 and Figure 5, when the opening angle decreased, the drag force and drag coefficient corresponding to the same velocity also decreased. Because the stroke angle decreased, the effective surface area of the stroke also decreased. In order to more clearly represent the resistance coefficient variation trend under different finger extension angles and different stroke extension angles, the calculated results were drawn into the following curve graph to make it easier to see their variation trend.

In summary, we found that the greater the angle of the five fingers, the lower the resistance value and the lower the resistance coefficient. It is recommended when swimming that one should take a hand stroke with the five fingers together as much as possible.

#### 3.2.2. Analysis of the Timing of Arm and Leg Coordination

**Figure 6:**
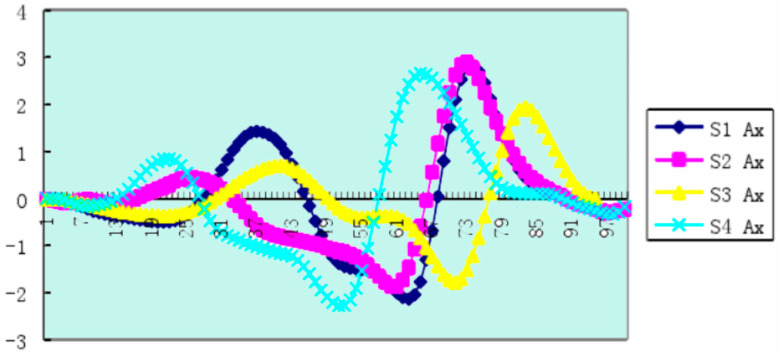
The horizontal speed graph of the center of gravity of the four athletes. The abscissa is the time of a swimming cycle, and the ordinate is in radians per second.

Based on the data available online, we visualized the data and made the change curve of the indicators of each part of the athlete’s body. We analyzed the images of the horizontal acceleration of the center of gravity of four athletes while swimming, brought the data into the effective dynamic model and then visualized the results of the calculations, getting the results shown in Figure 6.

Figure 6 shows that after the arm stroke, the horizontal speed of the center of gravity reached its maximum, and the speed had a relatively sharp drop, suggesting that the reason for this is the increase in resistance caused by the body lifting, which is related to dynamic factors [34].

We also found that for four athletes’ leg moments in the center of gravity, the rate of the first peak (maximum speed) before and after the athletes’ S1 and S3 moments and the center of gravity’s horizontal velocity maximum moment of the leg were almost the same, being only 0.02 s ahead of time, while for athletes S2 and S4, the biggest moment of closed legs in the center of gravity’s horizontal velocity moment had a time of 0.06 s. Reducting the legs early increased the drag time earlier, resulting in a faster rate of decline in the horizontal velocity of the center of gravity, or the center of gravity began to fall before reaching the maximum velocity.

Through this process, we can sum up the following law of arm–leg coordination: “Extend the arm first, then retract the leg”. Because the “first arm extension” is to maximize the propulsion [35] impulse of the rower and reach the maximum speed of the body, the “rear leg” is started when the body has moved to a higher extension position during the arm extension phase. This is quite helpful to reduce the resistance in exercise.

#### 3.2.3. Numerical Analysis of the Angular Velocity Variation of the Human Body

The size of the link angular velocity reflects the speed of the link’s rotation with small fluctuations and slow rotation, which has a positive effect on reducing the resistance of the body during exercise. The change of the link angular velocity can show the technical differences of the athletes.

**Figure 7:**
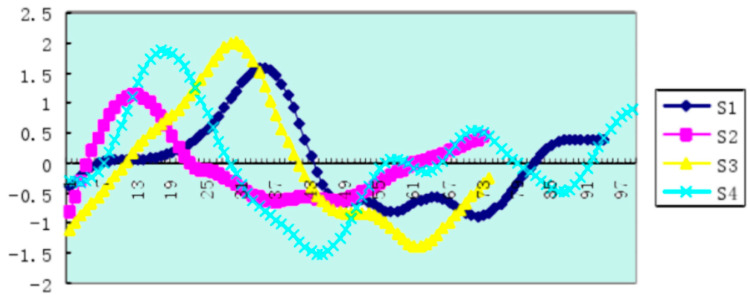
Angular velocity change curve of the sample athlete’s trunk links. The abscissa is the time of a swimming cycle, and the ordinate is in radians per second.

**Figure 8:**
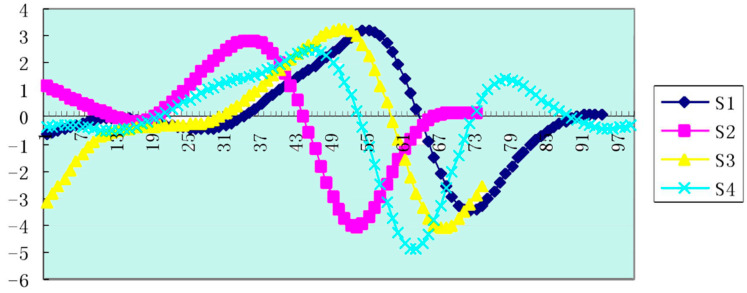
The variation curve of the angular velocities of the thighs of the sample athletes. The abscissa is the time of a swimming cycle, and the ordinate is in radians per second.

**Figure 9:**
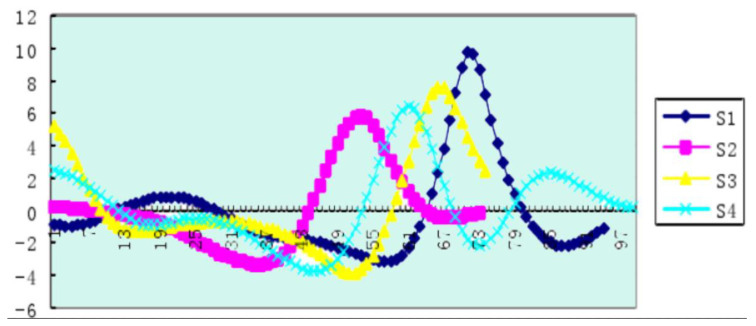
The curve of the angular velocity of the calves of the sample athletes. The abscissa is the time of a swimming cycle, and the ordinate is in radians per second.

Similarly, by collecting relevant materials, we could use effective dynamic models and visualize the data. Using Sbcas2 plane symmetry point motion analysis software, the data smoothing adopted cubic spline smoothing. The frequency of image acquisition and the velocity measurement were both 50 Hz, while 3D kinematic analysis adopted Simi image analysis software for areas such as one side of the hand, the wrist, elbow, shoulder, waist, hip, knee, ankle and heel. The key node was analyzed. We obtained the connecting rod angular velocity change curve of the athlete’s trunk, thigh and calf as shown in Figure 7, Figure 8 and Figure 9.

Athlete S1′s leg retraction technique better reflected the coordinated relationship between time and resistance. The benefits of rapid leg retraction surpass the speed loss caused by the increase in resistance. It finds a balance point in terms of speed loss and time saving from the center of gravity speed. The time between the maximum peak and the lowest valley (the second valley and the first peak) was the shortest in athlete S1, which was 0.46 s, and it was 0.54–0.60 s for the other athletes. Athlete S1 showed the shortest action time for changes in the angular velocity of the calf link and the biggest change.

### 3.3. Experiment

This part mainly introduces the measurement method and overall design scheme of the experimental data from Table 1, Table 2, Table 3, Table 4 and Table 5.

According to the purpose of this study and the technical route of the research scheme, the overall design of the experimental scheme needed to meet the requirements of the measurement of the static resistance and dynamic resistance of the human body in water, the analysis of two-dimensional and three-dimensional underwater image motion and the synchronous signal transmission and reception of force measurement, velocity measurement and image acquisition.

The force measuring system was connected by a series of devices, such as a force sensor, amplifier, AD conversion card and computer.

The speed measurement system included two methods: one was the speed control during force measurement, where the wire rope was driven by the frequency converter to provide a uniform traction speed, and the other was when the underwater image was collected. The speedometer measured the athlete’s level when swimming.

The underwater image acquisition was completed by the sealed camera lens (camera model: WAT-231D, made in Japan). In the dynamic resistance measurement, the underwater motion images of the athletes were tracked, taken and synchronized with the data collection of the force measurement system. When the underwater image was taken, it was synchronized with the data collection of the tachometer system.

**Figure 10:**
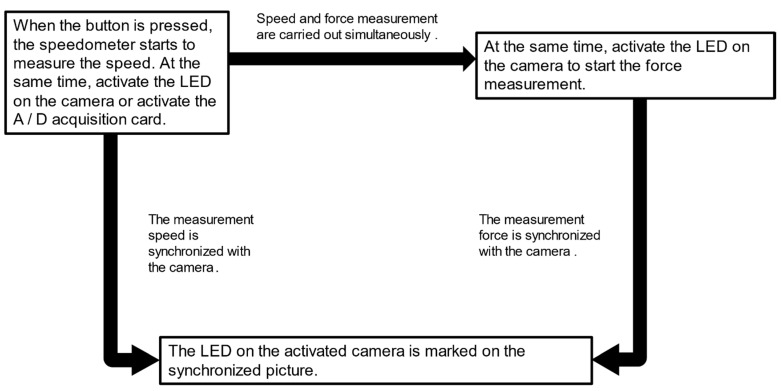
Schematic diagram of the experimental data testing process.

The experimental test control process is shown in Figure 10.

## 4. Discussion

Swimming is not only a widely developed sports and entertainment project, but it has also become a key project for countries to compete for advantages in competitive sports. In today’s increasingly fierce competitive swimming competition, it has become the consensus of coaches, athletes and scientific researchers to fully rely on science and technology to seek scientific training methods for improving athletes’ physical fitness and swimming skills. Among them, as far as swimming technology is concerned, formulating and exploring methods to reduce resistance and increase propulsion is a current research hotspot in the forefront of biomechanics. By combining this research, we will discuss the resistance in swimming (including static resistance and dynamic resistance) and the research of movement techniques.

Previous studies mainly focused on training effects and swimmers’ physical performance variables, such as oxygen uptake [36], biomechanical indicators (swimming length [9] and frequency) and propulsion efficiency [37]. In reality, however, the performance enhancement may be due to the active reduction of resistance [34]. In addition, the adjustment of body joint angles performed in this study may help to achieve better technical swimming performance at higher speeds. Therefore, for master swimmers, it seems to be an effective strategy [38] to develop their technical skills while conditionally and accurately training the details of their body movements. These improvements are more obvious when traveling long distances. Previous studies have been very thorough in the specific direction, but there is a lack of sufficient discussion on the macro posture and micro body movement details. Our research was based on the perspectives considered by the above researchers, and the participants were mainly swimmers and well-trained masters, which means their skill level can make greater progress. Therefore, their performance has a lot of potential to improve.

Based on the research situation at home and abroad, this research avoids the misunderstanding of being caught in research difficulties without practical results. Through the definition of propulsion and dynamic resistance, we analyzed the influence of the swimming technique on the propulsion and dynamic resistance so as to diagnose the movement technique in turn. The measurability of the dynamic resistance and propulsion and their decisive role in determining the swimming speed make this research and sports practice a feasible implementation plan, and at the same time, it is also a pioneering approach to swimming technology. The comprehensive analysis of kinematics and dynamics provides the basis.

In subsequent studies, some obvious limitations should be addressed, such as the insufficient sample size, more male swimmers than female swimmers and differences in competition experience. We also observed some factors of the internal variability of the subjects, such as the difference in physical strength consumed by different swimming styles. In addition, the proportional coefficient of resistance measurement was different among the 12 swimmers with different poses. Therefore, controlling more internal factors is also something to pay attention to in future research.

## 5. Conclusions

Based on the above research, if only from the point of view of reducing resistance, leg retraction should be completed at a slower speed and for a longer time. The rate of drag increase will be relatively slow, but there is also the effect of the propulsive force to be considered. From the perspective of saving movement time and improving movement speed, it is necessary to speed up the movement frequency, which is closely connected with the arm stroke, and the leg retraction should not be too negative, but it should be completed quickly. We explored the following results in the training of swimmers:

(1) From the numerical simulation results, it can be seen that at the same speed, the greater the angle of the five fingers, the lower the resistance value and the lower the resistance coefficient. According to the principle of force interaction, when the resistance is small, the propulsion is naturally small, which is detrimental to swimming. Therefore, it is recommended that one should engage in five-finger paddling in swimming.
(2) The speed was less than 0.5 m/s, but the maximum speed was generated in the rower stage. The thrust impulse of the kick plays a major role in increasing the speed of the human body by nearly 1 m/s. During training, athletes, especially long distance swimmers, should be taught to use as much energy as possible to kick if they have limited energy.
(3) After the arm stroke makes the horizontal speed of the center of gravity reach its maximum value, there is an obvious and relatively sharp drop in speed, suggesting that the reason for this is the increase in resistance caused by the body lifting.
(4) The timing of arm and leg coordination varies from person to person. Athletes should try to retract their legs after the horizontal speed of the person’s center of gravity reaches the maximum value after the stroke.
(5) According to the image of the angular velocity change of the torso, thigh and calf, the training direction of the athletes is mainly as follows: make the angular velocity fluctuation of the torso link as small as possible. Meanwhile, the angular velocity change of the calf link shows the characteristics of a slow start, short action time and great change.
(6) The horizontal velocity of the hip joint and the horizontal velocity of the center of gravity have a similar change rule and also show a dynamic curve. At the time of the characteristic value, it was earlier than the horizontal velocity of the center, and the values were also different. For example, at the end of the inner stroke, the horizontal velocity of the hip joint reached the maximum value, and the analytical result was 0.545–1.785 m/s. After the inner stroke, the maximum value of the horizontal velocity of the center of gravity was 1.338–1.553 m/s. The horizontal velocity of the hip joint was minimized at the time of maximum retraction.
(7) Therefore, the current technique puts forward the point of “late legs”, which emphasizes maintaining a high position of the body and maintaining a streamlined posture.

The conclusion drawn in the main body of this article was a velocity of 0.352–0.485 m/s. The minimum value of the horizontal velocity of the center of gravity was 0.481–0.823 m/s after the maximum leg was retracted. This shows that the horizontal velocity of the hip joint changed the proportion of gravity. The large changes in the cardiac horizontal velocity also had an important impact on the analysis of the athletes’ swimming postures. This factor should also be included in the analysis. However, due to the incompleteness of the data and the difficulty of the measurement and analysis, it was not included in the analysis. In future studies, this part should be included in the analysis, and the results will be clearer and more accurate.

## Figures and Tables

**Table 1:** Basic parameters of swimmers participating in the experiment.

Serial Number	Gender	Age (Year)	Height (m)	Weight (kg)	Sport Level
S1	Male	1981	1.88	94	International swimming master
S2	Male	1983	1.93	90	Swimming master
S3	Male	1986	1.76	71.5	Swimming master
S4	Male	1986	1.85	76	Swimming level 1
S5	Male	1985	1.83	75	Swimming master
S6	Male	1987	1.94	75	Swimming master
S7	Male	1988	1.71	71	Swimming master
S8	Male	1982	1.77	71	Athletics level 2
S9	Male	1959	1.78	70	Swimming level 3
S10	Male	1963	1.75	75	Athletics level 1
S11	Male	1969	1.74	59	Gymnastics level 2
S12	Female	1984	1.71	63	Swimming level 1
S13	Male	1985	1.78	65	Swimming level 1
S14	Female	1978	1.73	68	Swimming master
S15	Male	1981	1.80	70	Swimming master

**Table 2:** Experimental data of 15 athletes with different swimming styles (unit: degrees).

Aspect	Right Knee	Right Thigh	Right Leg	Five Fingers Open Angle	Type
S1	66.15	−47.26	−161.27	15	Breaststroke
S2	50.68	−55.83	−185.41	30	Freestyle
S3	64.23	−38.81	−152.11	25	Breaststroke
S4	53.17	−45.36	−172.83	20	Backstroke
S5	52.07	−43.58	−180.37	25	Butterfly
S6	65.29	−46.73	−163.78	20	Freestyle
S7	67.82	−45.69	−171.89	30	Breaststroke
S8	53.94	−39.51	−164.15	25	Breaststroke
S9	51.21	−43.83	−168.24	25	Backstroke
S10	63.44	−52.18	−158.92	15	Butterfly
S11	59.81	−51.72	−175.45	30	Breaststroke
S12	64.35	−47.91	−165.71	20	Backstroke
S13	56.99	−41.84	−149.16	25	Freestyle
S14	58.84	−49.93	−163.32	30	Freestyle
S15	66.13	−48.16	−179.27	25	Breaststroke

**Table 3:** Speed score ranking of athletes (unit: null).

	Distance to Best	Distance to Worst	Score	Rank
S1	0.863380710	0.165752736	0.442092808	10
S2	0.941091995	0.089763323	0.870765480	1
S3	0.740847903	0.262003560	0.426294448	11
S4	0.928943043	0.080153138	0.479430622	7
S5	0.872160719	0.129793015	0.529539928	5
S6	0.754065365	0.245522130	0.764970159	2
S7	0.800971412	0.282566941	0.410781669	12
S8	0.842769738	0.205629036	0.386914497	13
S9	0.882364429	0.197296868	0.462739595	8
S10	0.772873706	0.257444250	0.249868741	6
S11	0.586165198	0.673985766	0.361060489	14
S12	0.717438501	0.412332817	0.445623451	9
S13	0.756744825	0.245719093	0.745115150	3
S14	0.796854610	0.195110041	0.646690517	4
S15	0.749064606	0.301390918	0.296136281	15

**Table 4:** Score ranking of different swimmers based on the improved distance method (unit: null).

	Distance to Best	Distance to Worst	Score	Rank
S1	0.8691995	0.1657523	0.842092808	1
S2	0.9418943	0.0897633	0.487076548	9
S3	0.7408479	0.2620035	0.776294448	2
S4	0.9289430	0.0801301	0.379430622	13
S5	0.8721607	0.1297930	0.529539928	8
S6	0.7540653	0.2455221	0.464970159	10
S7	0.8009714	0.2825903	0.760781669	3
S8	0.8427603	0.2056290	0.686914497	4
S9	0.8823686	0.1972969	0.362739595	14
S10	0.7728737	0.2574442	0.549868741	7
S11	0.5861652	0.6739857	0.661060489	5
S12	0.7174766	0.4123328	0.445623451	11
S13	0.7567448	0.2457328	0.445115157	12
S14	0.7968546	0.1951178	0.646690517	6
S15	0.5496580	0.7013904	0.296136281	15

**Table 5:** Resistance and resistance coefficient scales corresponding to different five-finger tension angles and speeds.

Speed (m/s)	Five Fingers Open Angle							
	15°		20°		25°		30°	
	Resistance (N)	Resistance Coefficient	Resistance (N)	Resistance Coefficient	Resistance (N)	Resistance Coefficient	Resistance (N)	Resistance Coefficient
2.1	79.61	2.40	79.46	2.39	78.83	2.37	77.83	2.34
2.3	91.48	2.29	91.26	2.29	90.56	2.27	89.30	2.24
3.0	138.67	2.04	138.06	2.04	137.14	2.02	134.72	1.99
4.0	221.30	1.84	219.99	1.82	218.77	1.81	214.13	1.78
5.0	321.91	1.71	319.71	1.70	318.16	1.69	310.70	1.65

## Data Availability

Not applicable.
